# Administration of low-level laser on muscles of mastication following the induction of initial fatigue: protocol for a randomized, controlled, clinical trial

**DOI:** 10.1097/MD.0000000000011340

**Published:** 2018-06-29

**Authors:** Greice de Brito Bitencourt, Marcela Leticia Leal Gonçalves, Fernanda Yukie Kobayashi, Lara Jansiski Motta, Daniela Fátima Teixeira da Silva, Fabiano Politti, Leandro Paulino Feliciano, Raquel Agnelli Mesquita-Ferrari, Kristianne Porta Santos Fernandes, Sandra Kalil Bussadori

**Affiliations:** Universidade Nove de Julho, Liberdade, São Paulo, Brazil.

**Keywords:** initial fatigue, isometric contraction, low-level laser, masseter muscle

## Abstract

**Background:**

Orofacial pain encompasses painful conditions, such as temporomandibular disorder (TMD). Multidisciplinary health teams seek to control such musculoskeletal disorders to improve the quality and functional capacity of the muscles of mastication. The aim of the proposed study is to evaluate the effect of low-level laser therapy as a form of treatment for the prevention of initial fatigue of the muscles of mastication (masseter and anterior temporal muscles) as well as the recovery of these muscles after induced exhaustion (caused by isometric contraction) in young adults.

**Methods:**

The participants will be 78 healthy male and female volunteers between 18 and 34 years of age. The volunteers will be randomly allocated to a laser group (n = 26), sham group (n = 26), and control group (n = 26). All participants will be submitted to a clinical evaluation to record mandibular movements, bite force, muscle sensitivity to palpation, and initial muscle fatigue. Initial fatigue will be induced by isometric contraction of the jaws. Maximum voluntary contraction will be performed to record the time until initial exhaustion of the masseter muscle (determined by electromyography). The groups will then be submitted to the interventions: active laser therapy (wavelength: 780 nm; fluence: 134 J/cm^2^; power: 50 mW; irradiance: 1.675 W/cm^2^; exposure time: 80 seconds per point) on 3 points of the masseter and 1 point on the anterior temporal muscles on each side; sham laser (placebo effect); or no intervention (control). Maximum voluntary contraction will be performed again after the interventions to record the time until initial exhaustion of the masseter muscle (determined by electromyography). Differences in individual time until exhaustion between the pre- and postintervention evaluations will be measured to determine the effect of low-level laser therapy.

**Discussion:**

Although studies have been made with the use of low-level laser therapy in TMDs and on the effect of photobiomodulation on fatigue, this the first study to test this therapy in the prevention of fatigue in this region. The clinical relevance lies in the fact that longer dental procedures could take place if the patients are less prone to fatigue.

## 1 Introduction

Orofacial pain encompasses painful conditions of the mouth and face, such as temporomandibular disorder (TMD), which is defined as a set of painful or dysfunctional conditions that involve the muscles of mastication and the temporomandibular joint (TMJ).^[1]^ The masseter muscle has been found to be more susceptible to fatigue in certain adverse health conditions, such as TMD, chronic headache and neck injuries in some studies but is reported to be unaffected in other studies.^[2]^

Low-level laser therapy has been increasingly administered in recent years in an attempt to delay the process of muscle fatigue and improve muscle performance. Experiments performed with animals using red (655 nm) and infrared (904 nm) wavelengths demonstrate positive results in the improvement of muscle performance and studies involving human subjects report improvements with regard to strength training and recovery from exhaustion.^[3]^ Biostimulation with low-level laser is reported to have a set of positive effects, such as the acceleration of the healing of bone defects in vivo and in vitro, the stimulation of blood flow, the recruitment and activation of osteoblasts, the promotion of osteosynthesis, and a reduction in osteoclast activity as well as anti-inflammatory action.^[1,4]^

Surface electromyography (EMG) is used in the study of bioelectrical phenomena that occur in skeletal muscle fibers at rest as well as during effort and maximum contraction.^[5]^ EMG is a noninvasive technique that is easy to execute and enables the determination of the electrophysiologic behavior of muscles in different physiologic conditions. It can therefore assist in the detection of muscle fatigue. Surface EMG has been widely used by physicians, speech therapists, physiotherapists, and physical educators for the study of human movement.^[6]^ EMG clearly confirms and can quantify the presence and severity of muscular electrical dysfunction. Levels of EMG activity demonstrate the extent to which muscles are electrically active. Surface EMG is used in clinical practice, research, sports science, and other distinct fields. The use of EMG together with other clinical methods enables a better understanding of the electroneurophysiologic action of muscles.

The aim of the proposed study is to evaluate the effects of phototherapy on the muscles of mastication, on bite force, the range of mandibular movements, sensitivity to palpation, and time to exhaustion of the masseter and anterior temporal muscles when administered prior to the induction of muscle fatigue.

## 2 Materials and methods

### 2.1 Type of study

A randomized, blind, clinical trial will be conducted with both placebo and control groups. The guidelines for research involving human subjects stipulated in Resolution 466/2012 of the Brazilian National Board of Health will be followed and the study has been approved by the Human Research Ethics Committee of University Nove de Julho, under process number 76402417.2.0000.5511. The participants will sign a statement of informed consent after receiving clarifications regarding the objectives and procedures of the study.

**Figure 1:**
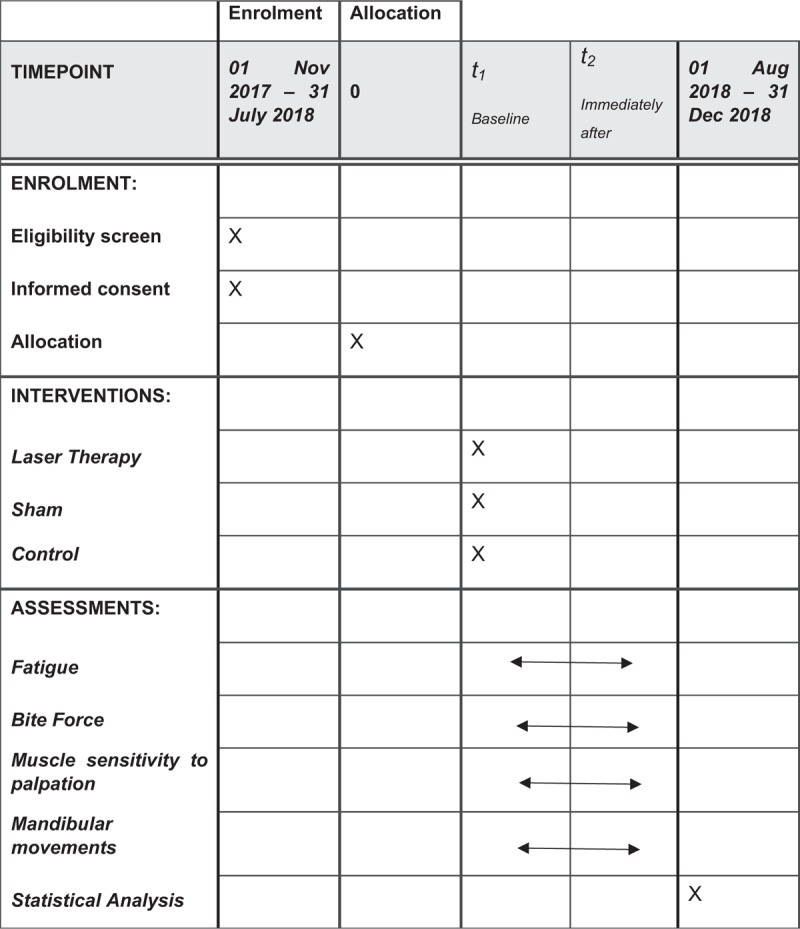
Standard Protocol Items: Recommendations for Interventional Trials (SPIRIT) figure as recommended by 2013 SPIRIT statement.

The protocol is in accordance with the 2013 SPIRIT (Standard Protocol Items: Recommendations for Interventional Trials) Statement. The SPIRIT checklist can be found as an additional file and Figure 1 is the SPIRIT figure. SPIRIT was developed to provide guidance in the form of a checklist of recommended items to include in a clinical trial protocol, to help improve its content and quality.

### 2.2 Trial registration

Clinicaltrials.gov as NCT03460639, first posted March 9, 2018; https://clinicaltrials.gov/ct2/show/record/NCT03460639

### 2.3 Sample calculation

The G∗ Power software (v.3.1.9.2, Franz Faul, Universität Kiel, Germany) was used for the calculation of the sample size, considering 3 groups (active laser, sham laser, and control) that will be evaluated before and after the interventions. To detect mean differences, 26 volunteers will be needed for each group (total: 78 volunteers).

Inclusion criteria: age 18 to 34 years, absence of TMD, and signed statement of informed consent.

Exclusion criteria: currently undergoing orthodontic or orthopedic treatment of the jaws, psychologic treatment or physical therapy, currently taking muscle relaxant or anti-inflammatory agent or using a bite plate.

### 2.4 Recruitment and randomization

**Figure 2:**
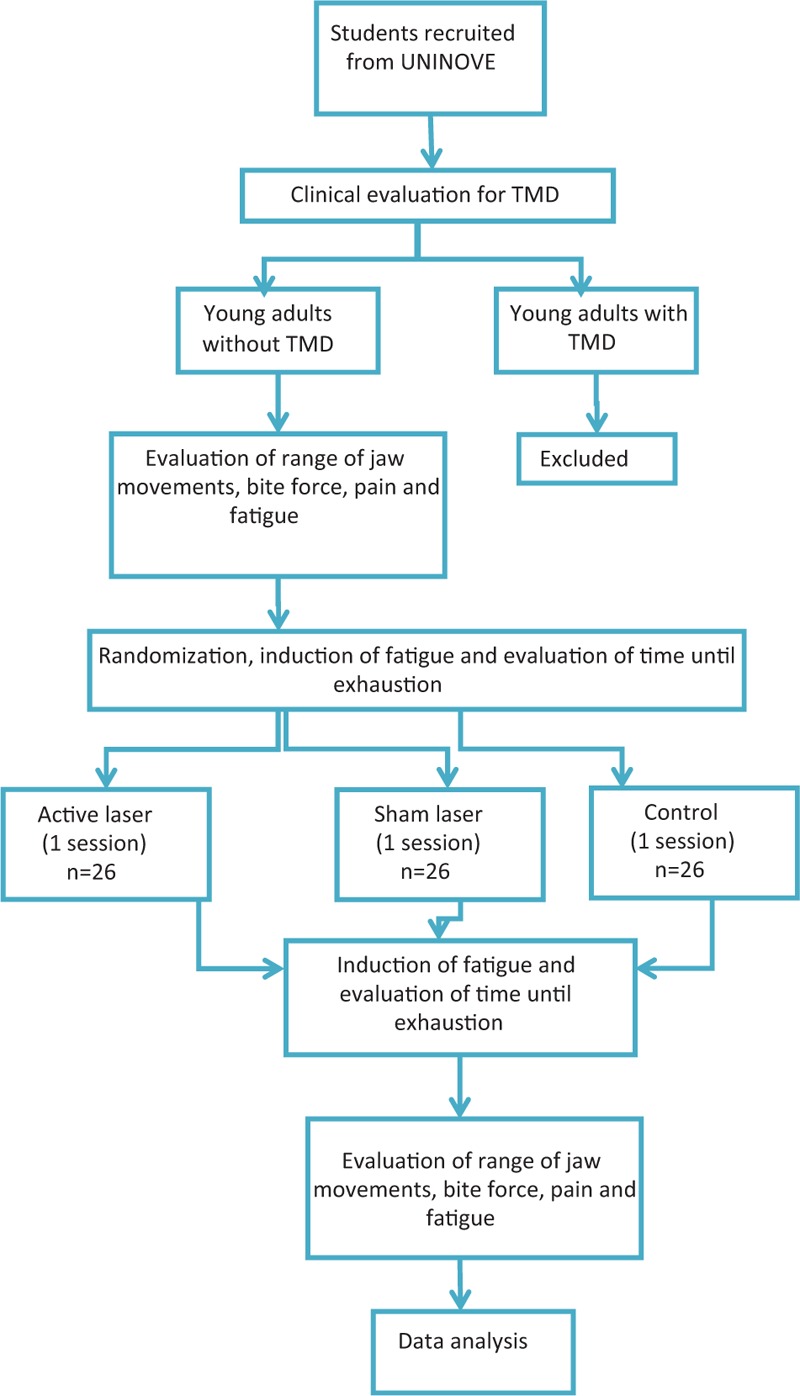
Flowchart activity.

Healthy male and female young adults enrolled at University Nove de Julho (Vergueiro campus) in São Paulo will be evaluated (Fig. 2). Since they will already be on campus, the process of recruitment will be simple. Patients will be divided by block randomization into the groups.

## 3 Outcomes

### 3.1 Induction of fatigue and EMG

The volunteer will be seated on a chair with back supported, eyes open, feet parallel on a rubber mat, and hands resting on thighs. The examination will be performed under 2 conditions: in normal postural position with mandible at rest; during maximum voluntary contraction (MVC) with Parafilm M placed between the teeth.

In the resting position, the volunteer will remain with the jaw relaxed, the teeth separated and the lips in contact for 15 seconds. For MVC, the volunteer will be instructed to place a Parafilm M between the maxillary and mandibular premolars and second molars bilaterally and perform isometric contraction for 45 seconds after a verbal command.

A contraction protocol will be used to induce fatigue.^[7]^ MVC clenching the teeth will be performed to record the time until initial exhaustion of the masseter muscle (determined by EMG). The groups will then be submitted to the interventions and MVC will be performed a second time to record the time until initial exhaustion of the masseter muscle (determined by EMG). During the procedures, the volunteers will receive verbal encouragement as well as visual feedback on the monitor to maintain MVC. Before the readings, the volunteers will undergo training of the procedure for the acquisition of the EMG signal.^[8]^

### 3.2 Laser application protocol

Photobiomodulation will be performed with the Twin Flex Evolution device from MM Optics (São Carlos, SP, Brazil). The protocol proposed by Godoy et al^[9]^ for the control of facial muscle pain will be employed. This protocol was used in a previous study and proved effective when 12 consecutive applications were performed with the aim of evaluating the immediate effect of irradiation.^[4]^

The volunteer will be seated in a quiet environment without sound interference and positioned with the Frankfurt plane parallel to the floor. The active tip of the laser will be covered with plastic wrap to avoid cross-contamination and for reasons of hygiene. The sites to be irradiated will first be cleaned with 70% alcohol.

Three points on the masseter muscle (upper, middle, and lower portions) and 1 point on the anterior temporal on each side of the face will be irradiated with a wavelength of 780 nm, radiant exposure of 134 J/cm^2^, power of 50 mW and irradiance of 1.675 W/cm^2^ for 80 seconds per point, resulting in an energy of 4 J per point and total energy of 32 J per volunteer.^[4,9]^ Point application will be performed with a conventional tip in contact with the skin (beam spot: 0.04 cm^2^). All parameters can be found in Table 1.

**Table 1:**
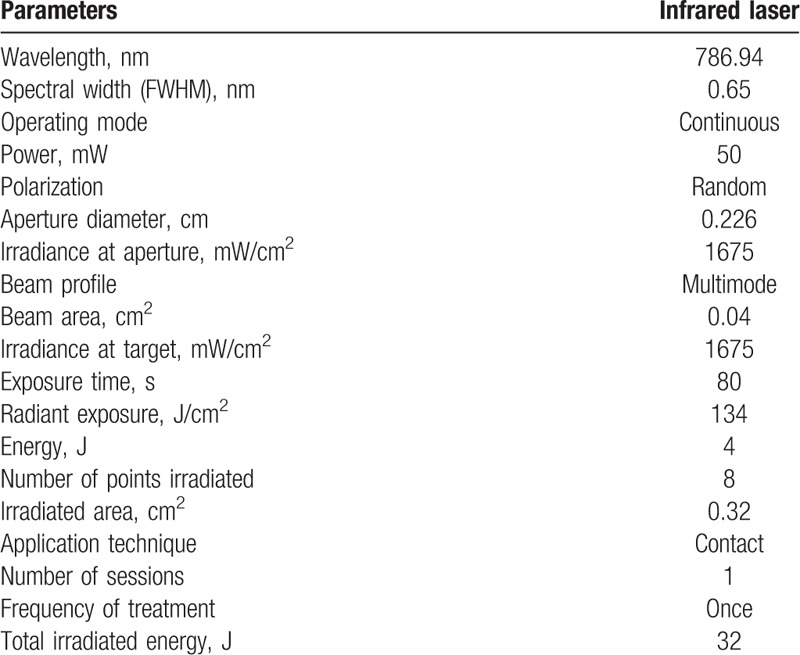
Laser parameters.

The same procedures will be performed in the sham group, but the device will be switched off and a recording of the emission sounds will be used to give the volunteer the auditory sensation of laser therapy.

### 3.3 Research diagnostic criteria for TMD

The research diagnostic criteria for temporomandibular disorder (RDC-TMD) questionnaires will be filled out by the volunteers for the diagnostic investigation of TMD. A specific clinical examination will be performed always by the same previously trained evaluator and will consist of palpation of the trapezius, sternocleidomastoid, temporal, masseter, digastrics and medial pterygoid muscles, palpation of the TMJs, analysis of mandibular movements (vertical and horizontal movements measured with digital calipers), the verification of joint sounds and the investigation of frequent headaches, facial pain, fatigue and difficulty chewing, bruxism, psychologic aspects, and parafunctional habits. Volunteers classified in groups I, II, or III of the RDC-TMD will be excluded from the study.

### 3.4 Bite force

The DMD digital dynamometer (Kratos Equipamentos Industriais Ltda, Cotia, SP, Brazil) will be used to measure bite force. This is an electronic device composed of a bite fork and digital display connected by a wire. Readings will be taken with the fork in the region of the first molars and the data will be recorded in kilograms of force. The participants will receive instructions and training prior to the readings. Six readings will be made (3 on each side), alternating sides between readings. Each reading will last 5 seconds, with a 1-minute rest between readings.^[10]^ Bite force will be measured before and after the interventions.

### 3.5 Range of mandibular movements

For the evaluation of the range of mandibular movements, we will follow the guidelines of the International Association for Dental Research for the use of the RDC-TMD, with a single measurement of each movement.^[11]^ The volunteer will be instructed to open his/her mouth as wide as possible for the measurement of maximum voluntary vertical mandibular movement with the aid of digital calipers (distance between maxillary and mandibular central incisors). The volunteer will then repeat the movement and the calipers will exert pressure against the maxillary and mandibular incisors to obtain maximum passive vertical mandibular movement. These procedures will be performed at the initial and final evaluations to record the range of mandibular movements before and after the interventions.

### 3.6 Pain evaluation

The visual analog scale will be used to record pain upon palpation of the masseter and anterior temporal muscles. This is a numeric scale that ranges from 0 (absence of pain) to 10 (most intense pain).^[12]^ The scale will be used at the initial and final evaluations to record pain before and after the interventions.

### 3.7 Statistical analysis

The results will be submitted to statistical analysis. The Shapiro–Wilk test will be used to determine adherence to the Gaussian curve. Parametric data will be compared using repeated-measures analysis of variance and nonparametric data will be compared using the Kruskal–Wallis test. A *P* < .05 will be considered indicative of statistical significance.

## 4 Discussion

Although studies have been made with the use of low-level laser therapy in TMD and on the effect of photobiomodulation on fatigue,^[13–16]^ this the first study to test this therapy in the prevention of fatigue in this region. The clinical relevance lies in the fact that longer dental procedures could take place if the patients are less prone to fatigue.

## 5 Declarations

### 5.1 Ethics committee

The Ethics Committee of University of Nove de Julho (UNINOVE) approved this project under process number 76402417.2.0000.5511, in accordance with the guidelines of the National Ethics Committee (CONEP). Any modifications to the protocol that may have an impact on the conduct of the study will be reported to the committee. An informed consent form (which was approved by the UNINOVE Ethics Committee) will be signed by the volunteers previous to their participation.

### 5.2 Data collection methods

The authors were previously trained to collect data and perform the evaluations. All authors are qualified in laser therapy. The same researchers will enroll patients and assign them to interventions. All data will be entered electronically. The participants’ files will be stored in numerical order in a safe place and accessible only to the authors of this study.

### 5.3 Discontinuing intervention

If volunteers become ill or do not adapt to therapy, it will not be possible to continue laser therapy. Interventions will be made while the participants are at the university, to make it easier for them to come, avoiding absences. No adverse effects are expected.

### 5.4 Availability of data and materials

The data sets generated and analyzed during the present study are available from the corresponding author at reasonable request. After the analysis of the data, volunteers will be invited to a meeting and the results will be shared and they will become public.

## Author contributions

Conceive and design the study: Greice de Brito Bitencourt, Sandra Kalil Bussadori; will perform the experiment: Greice Brito, Fabiano Politti, Leandro Paulino Feliciano; will analyze the data: Fernanda Yukie Kobayashi, Lara Jansiski Motta, Daniela Fátima Teixeira da Silva, Raquel Agnelli Mesquita-Ferrari, Kristianne Porta Santos Fernandes, Sandra Kalil Bussadori; will perform the statistical analysis: Lara Jansiski Motta, Daniela Fátima Teixeira da Silva; write the paper: Greice Brito, Marcela Leticia Leal Gonçalves, Sandra Kalil Bussadori.

**Conceptualization:** Greice Brito, Fernanda Yukie Kobayashi, Sandra Kalil Bussadori.

**Data curation:** Raquel Agnelli Mesquita-Ferrari.

**Formal analysis:** Lara Jansiski Motta, Daniela Fátima Teixeira da Silva.

**Investigation:** Greice Brito, Fernanda Yukie Kobayashi, Fabiano Politti, Leandro Paulino Feliciano.

**Methodology:** Greice Brito, Fernanda Yukie Kobayashi, Fabiano Politti, Leandro Paulino Feliciano.

**Project administration:** Greice Brito, Raquel Agnelli Mesquita-Ferrari, Kristianne Porta Santos Fernandes, Sandra Kalil Bussadori.

**Resources:** Kristianne Porta Santos Fernandes.

**Software:** Marcela Leticia Leal Gonçalves, Lara Jansiski Motta, Daniela Fátima Teixeira da Silva.

**Supervision:** Greice Brito, Sandra Kalil Bussadori.

**Validation:** Greice Brito, Marcela Leticia Leal Gonçalves, Sandra Kalil Bussadori.

**Visualization:** Greice Brito, Marcela Leticia Leal Gonçalves, Fernanda Yukie Kobayashi, Lara Jansiski Motta, Daniela Fátima Teixeira da Silva, Fabiano Politti, Leandro Paulino Feliciano, Raquel Agnelli Mesquita-Ferrari, Kristianne Porta Santos Fernandes, Sandra Kalil Bussadori.

**Writing – original draft:** Greice Brito, Fernanda Yukie Kobayashi, Lara Jansiski Motta, Fabiano Politti, Leandro Paulino Feliciano.

**Writing – review & editing:** Marcela Leticia Leal Gonçalves, Daniela Fátima Teixeira da Silva, Raquel Agnelli Mesquita-Ferrari, Kristianne Porta Santos Fernandes, Sandra Kalil Bussadori.
